# Supplementary material for: A New Mild Method for Synthesis of Marine Alkaloid Fascaplysin and Its Therapeutically Promising Derivatives
Source: Mar Drugs. 2023 Jul 25;21(8):424. doi: 10.3390/md21080424 (PMC10455802; doi:10.3390/md21080424)

Supporting information

A New Mild Method for Synthesis of Marine Alkaloid Fascaplysin and Its Therapeutically Promising Derivatives

Oleg A. Tryapkin [1,*], Alexey V. Kantemirov [1], Sergey A. Dyshlovoy [2,3], Vladimir S. Prassolov [4,5], Pavel V. Spirin [4,5], Gunhild von Amsberg [2,3], Maria A. Sidorova [1] and Maxim E. Zhidkov [1,*]

[1] Department of Chemistry and Materials, Institute of High Technologies and Advanced Materials, FEFU Campus, Far Eastern Federal University, Ajax Bay 10, Russky Island, 690922 Vladivostok, Russia; kantemirov_av@dvfu.ru (A.V.K.); sidorova_ma@dvfu.ru (M.A.S.)

[2] Department of Oncology, Hematology and Bone Marrow Transplantation with Section Pneumology, Hubertus Wald Tumorzentrum—University Cancer Center Hamburg (UCCH), University Medical Center Hamburg-Eppendorf, Martinistrasse 52, 20246 Hamburg, Germany; s.dyshlovoy@uke.de (S.A.D.); g.von-amsberg@uke.de (G.v.A.)

[3] Martini-Klinik Prostate Cancer Center, University Hospital Hamburg-Eppendorf, Martinistrasse 52, 20246 Hamburg, Germany

[4] Department of Cancer Cell Biology, Engelhardt Institute of Molecular Biology, Russian Academy of Sciences, Vavilova 32, 119991 Moscow, Russia; prassolov45@mail.ru (V.S.P.); spirin.pvl@gmail.com (P.V.S.)

[5] Center for Precision Genome Editing and Genetic Technologies for Biomedicine, Engelhardt Institute of Molecular Biology, Russian Academy of Sciences, Vavilova 32, 119991 Moscow, Russia

* Correspondence: triapkin_oa@dvfu.ru (O.A.T.); zhidkov.me@dvfu.ru (M.E.Z.)

Contents

Spectra Data 3-22

Spectra Data

^{1}H NMR spectra of 3-(3,3-dimethyl-2-oxobutyl)-3-hydroxyindolin-2-one

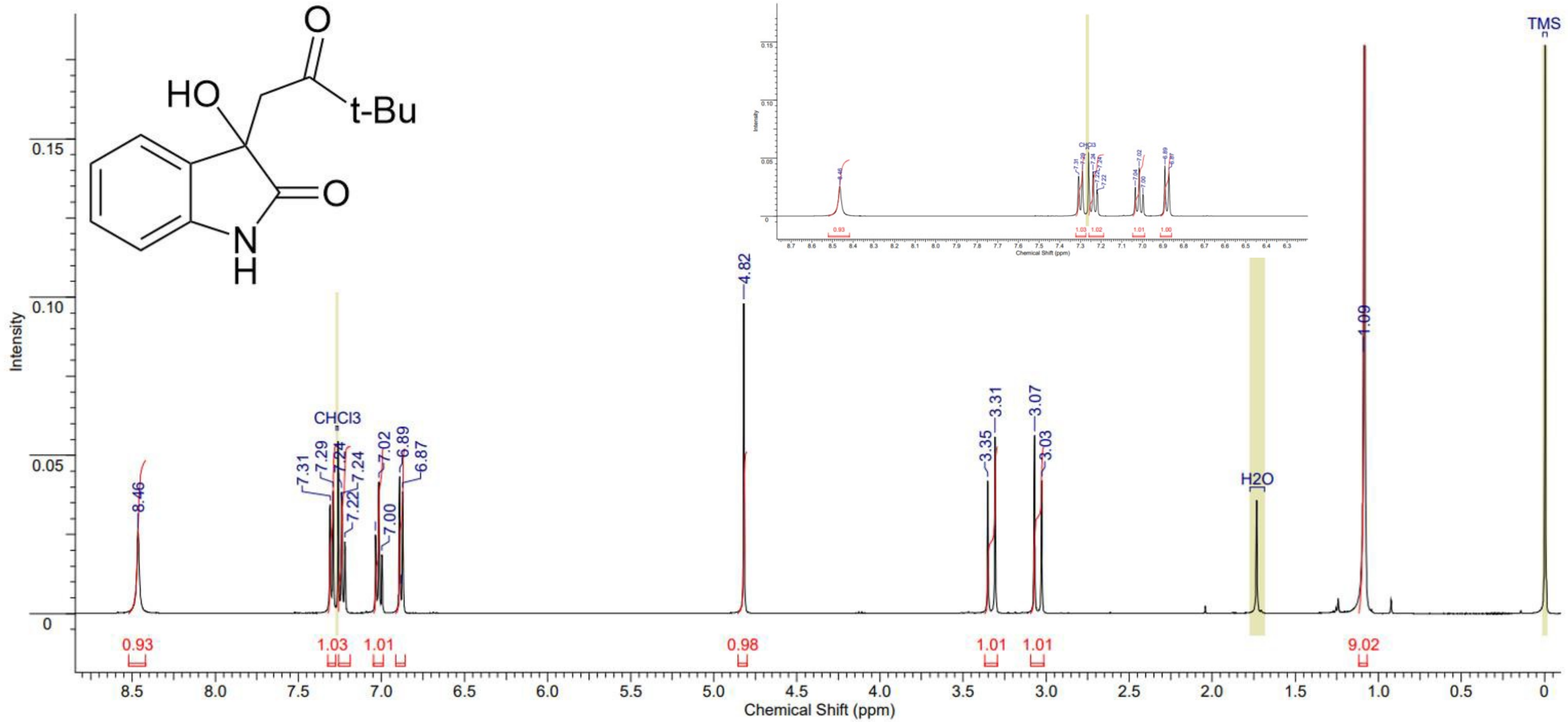

^{13}C NMR spectra of 3-(3,3-dimethyl-2-oxobutyl)-3-hydroxyindolin-2-one

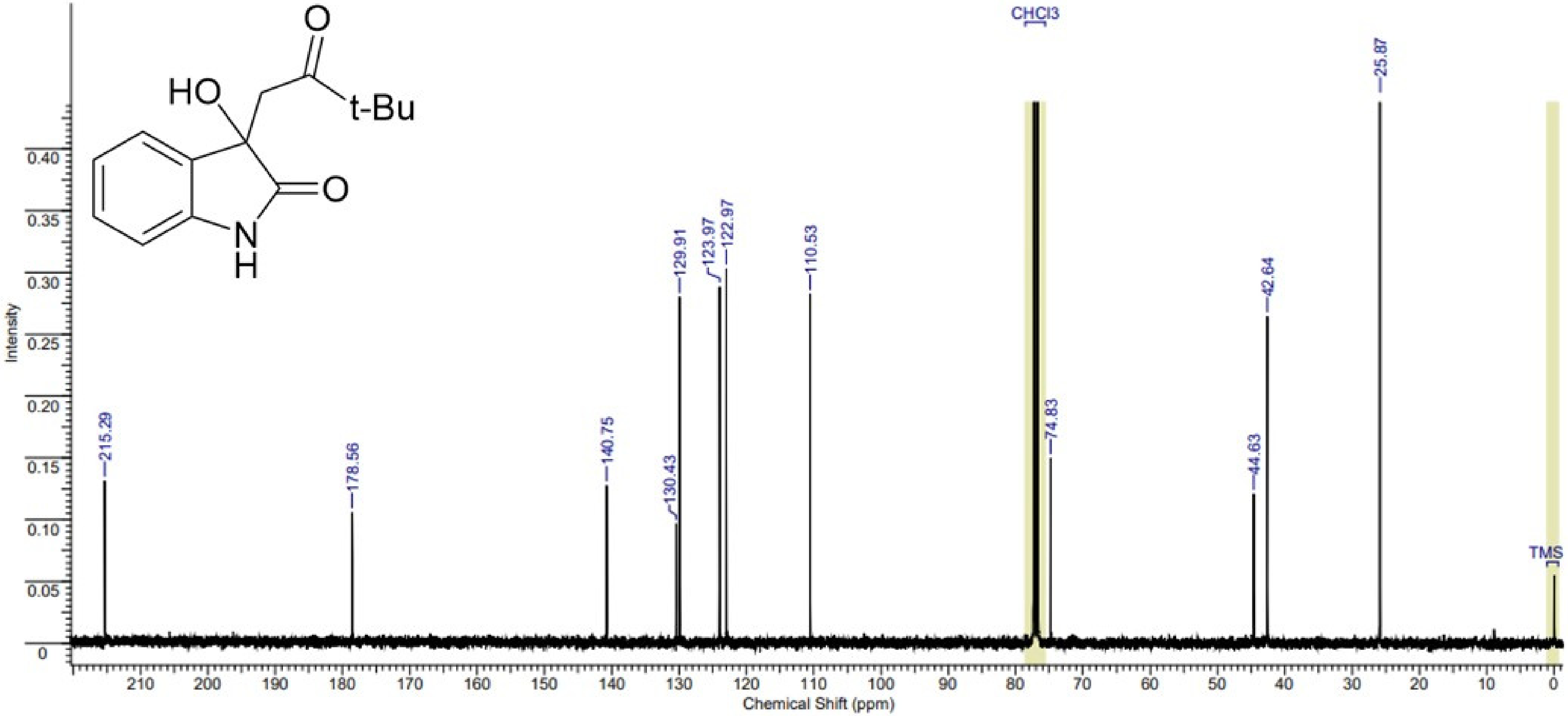

^{1}H NMR spectra of 3-(3,3-dimethyl-2-oxobuten-4-yl)-indolyn-2-one

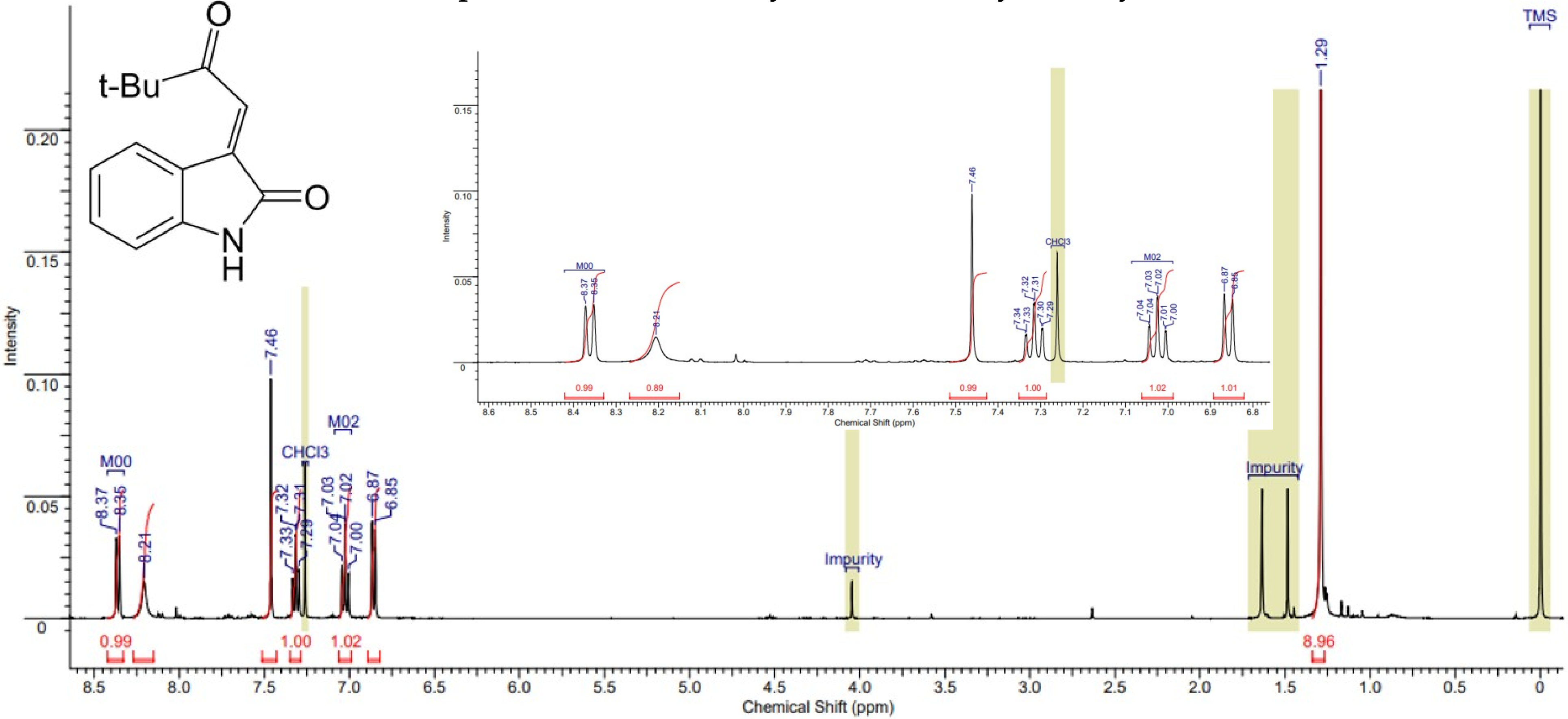

^{13}C NMR spectra of 3-(3,3-dimethyl-2-oxobuten-4-yl)-indolyn-2-one

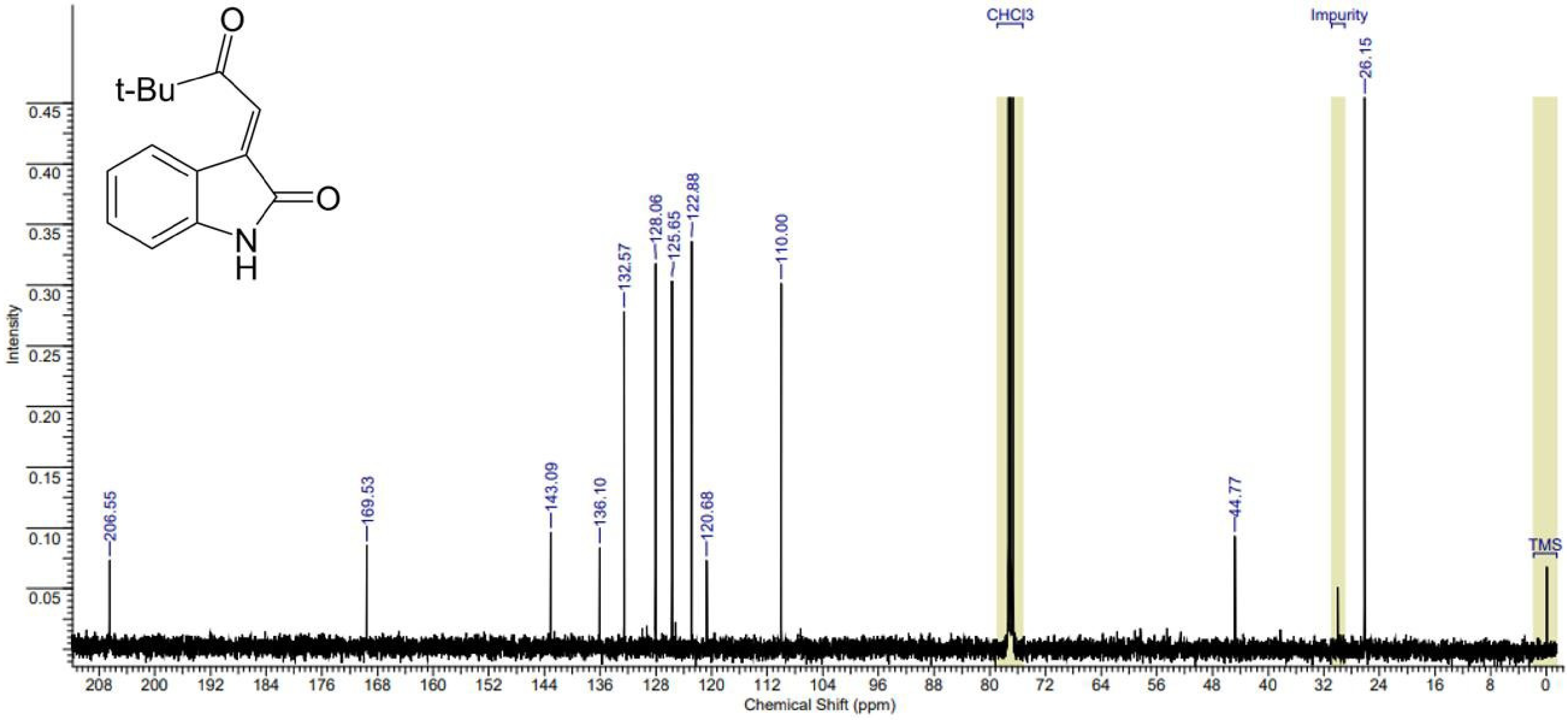

^{1}H NMR spectra of 3-(3,3-dimethyl-2-oxobutyl)-indolyn-2-one

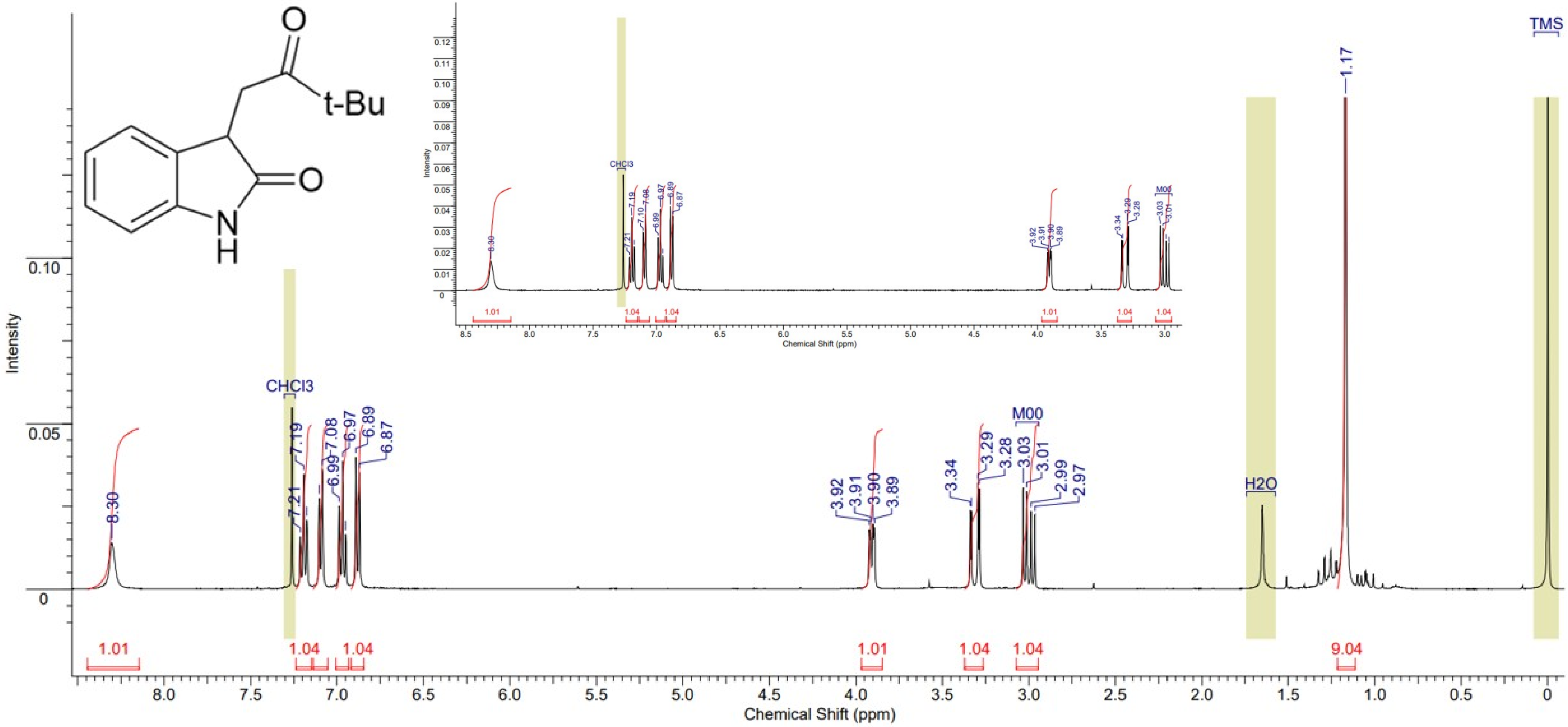

^{13}C NMR spectra of 3-(3,3-dimethyl-2-oxobutyl)-indolyn-2-one

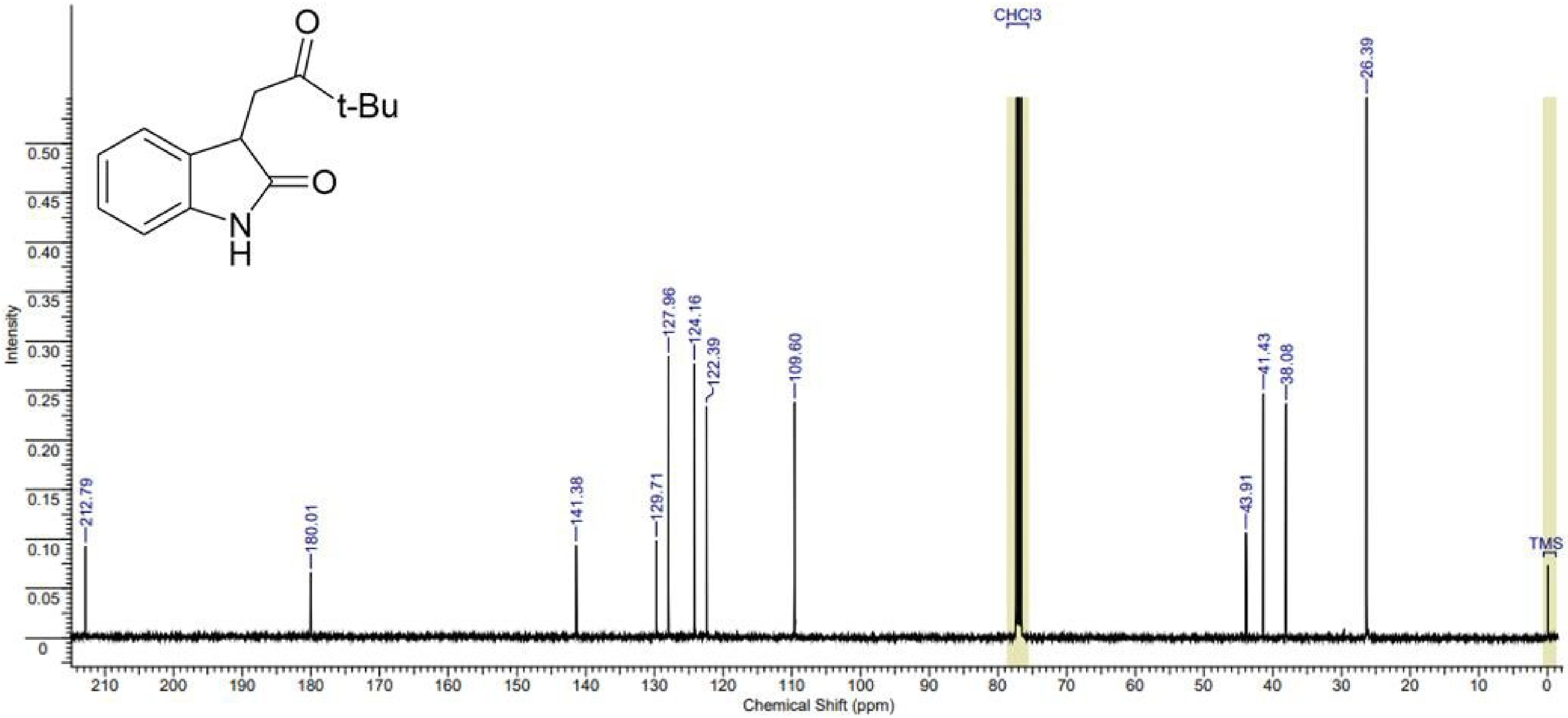

^{1}H NMR spectra of 3-(2-(hydroxyamino)-3,3-dimethylbutyl)-indolyn-2-one

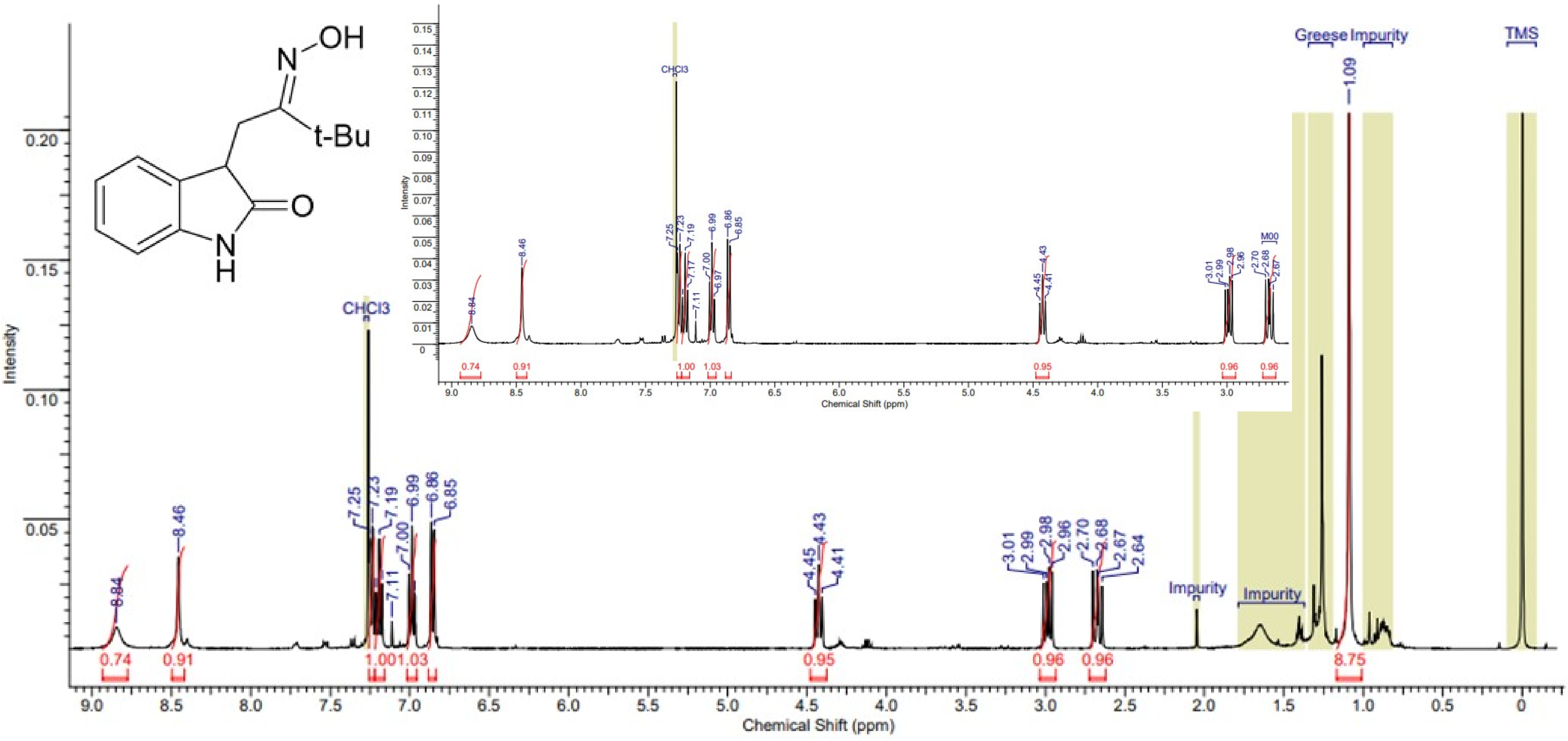

^{13}C NMR spectra of 3-(2-(hydroxyamino)-3,3-dimethylbutyl)-indolyn-2-one

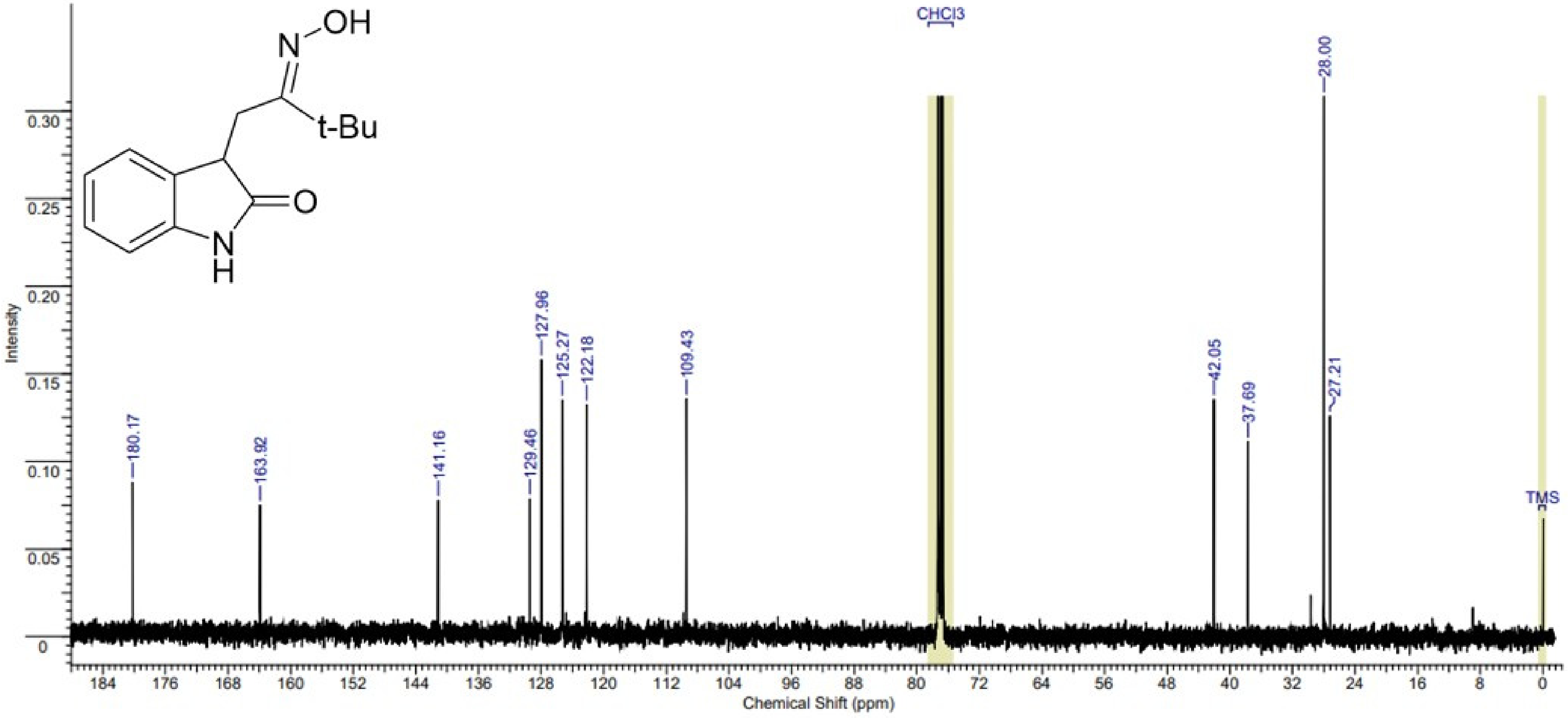

^{1}H NMR spectra of 1-(2′-bromobenzoyl)-6-benzyloxy-β-carboline

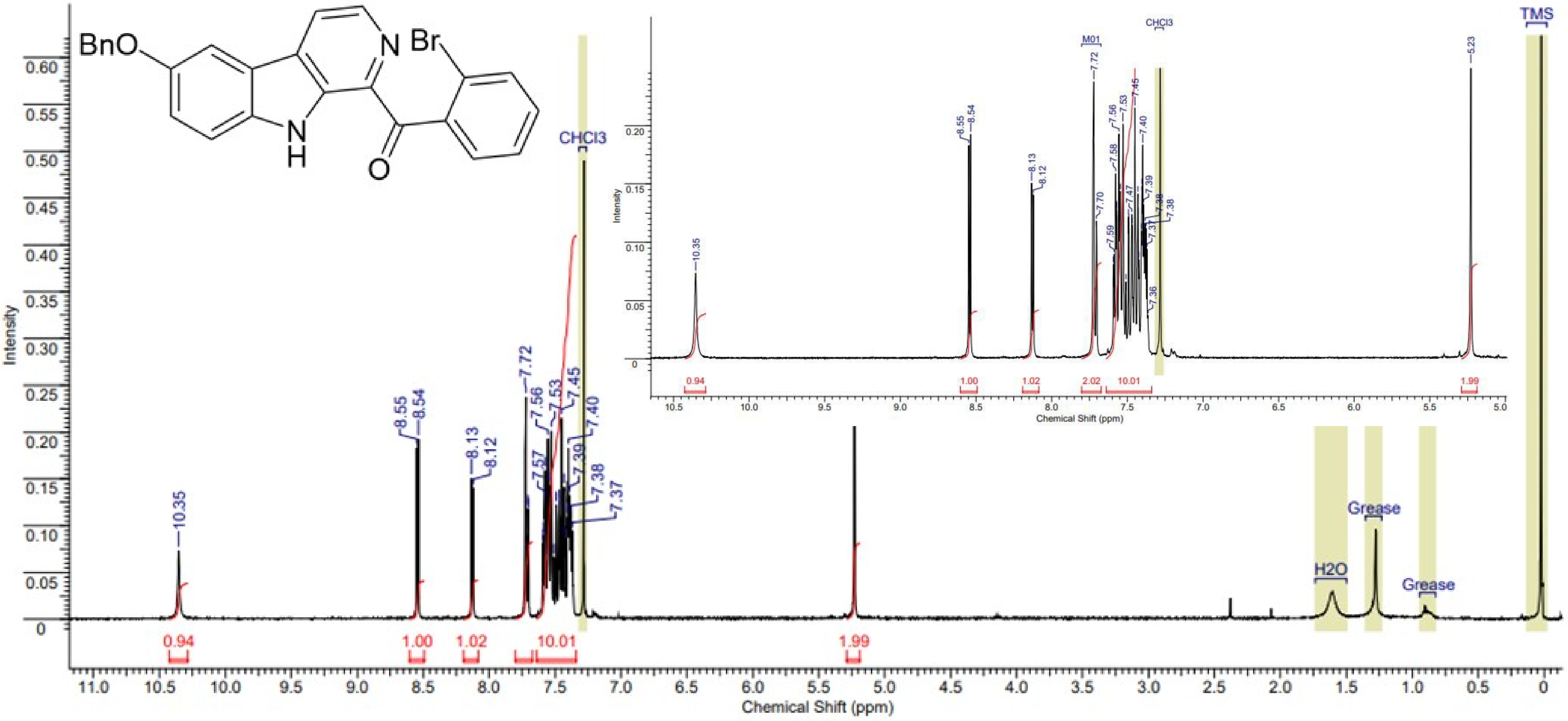

^{13}C NMR spectra of 1-(2′-bromobenzoyl)-6-benzyloxy-β-carboline

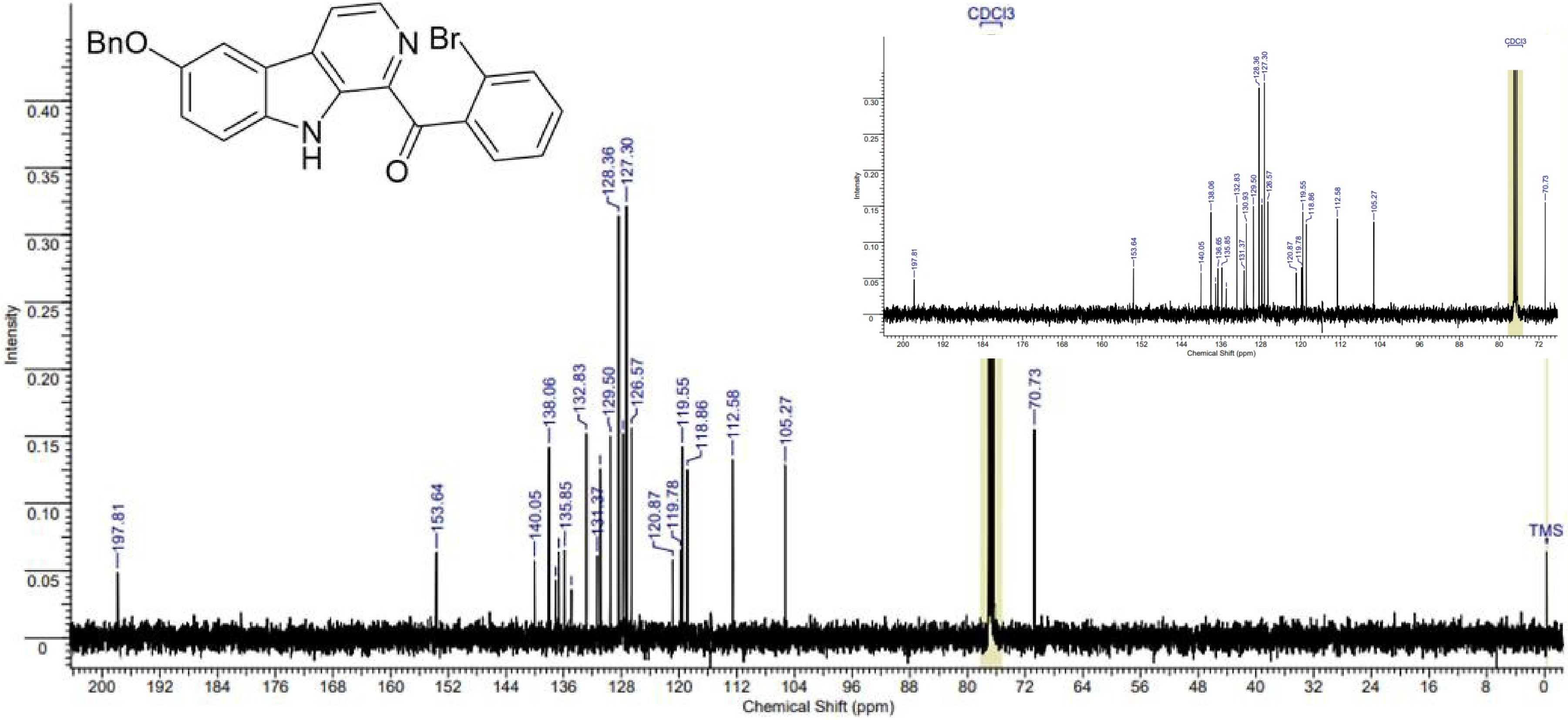

^{1}H NMR spectra of 1-(2′-iodobenzoyl)-6-benzyloxy-β-carboline

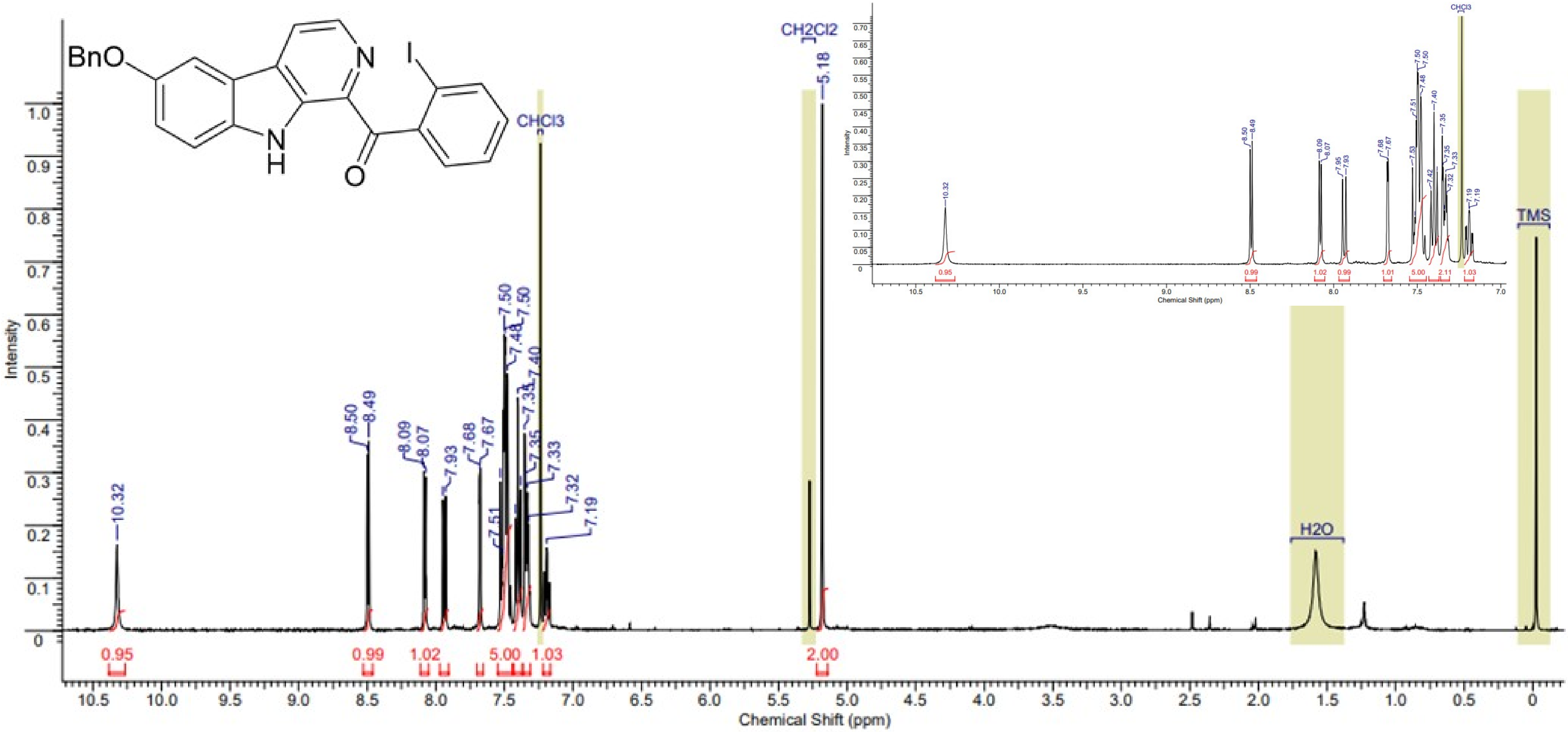

^{13}C NMR spectra of 1-(2′-iodobenzoyl)-6-benzyloxy-β-carboline

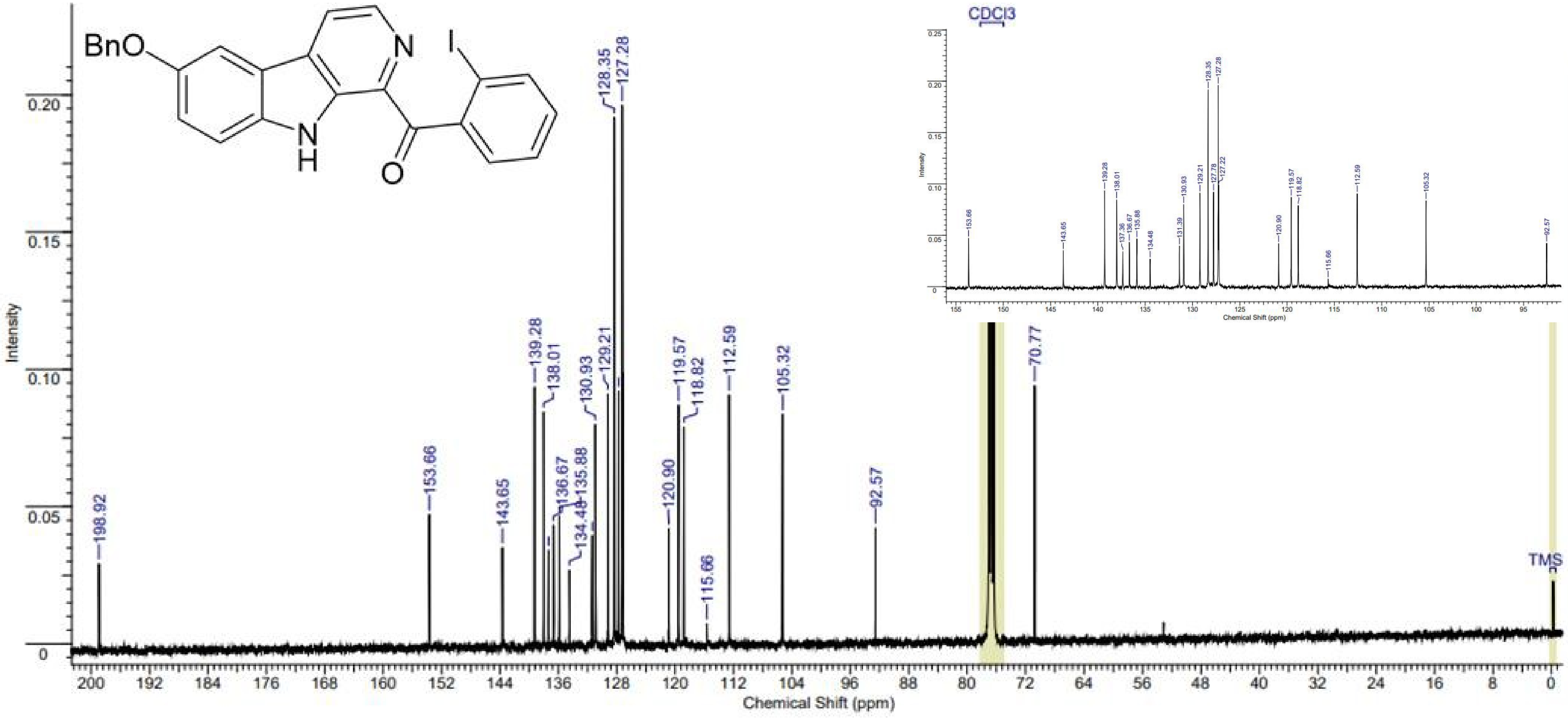

^{1}H NMR spectra of 1-(2′-bromobenzoyl)-3-*tert*-butyl-β-carboline

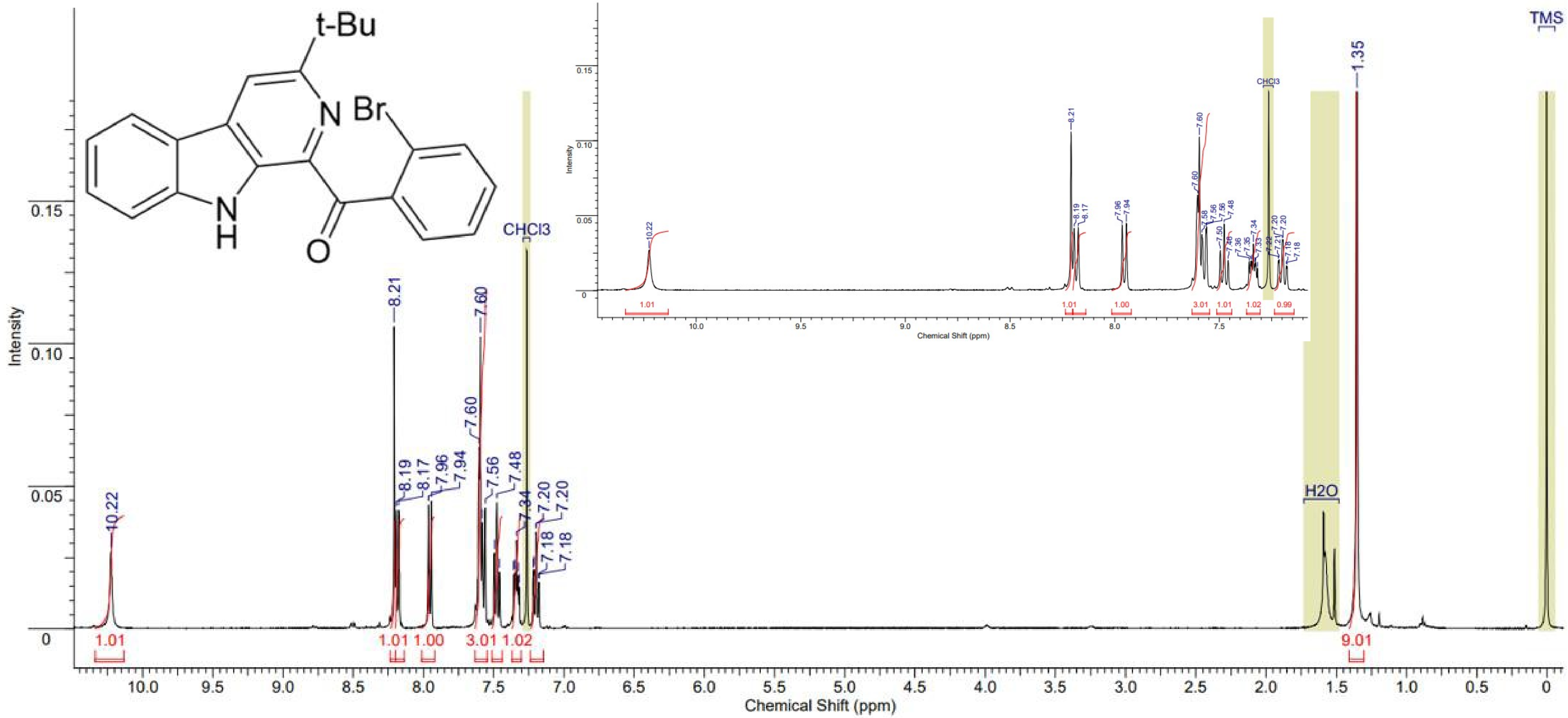

^{13}C NMR spectra of 1-(2′-bromobenzoyl)-3-*tert*-butyl-β-carboline

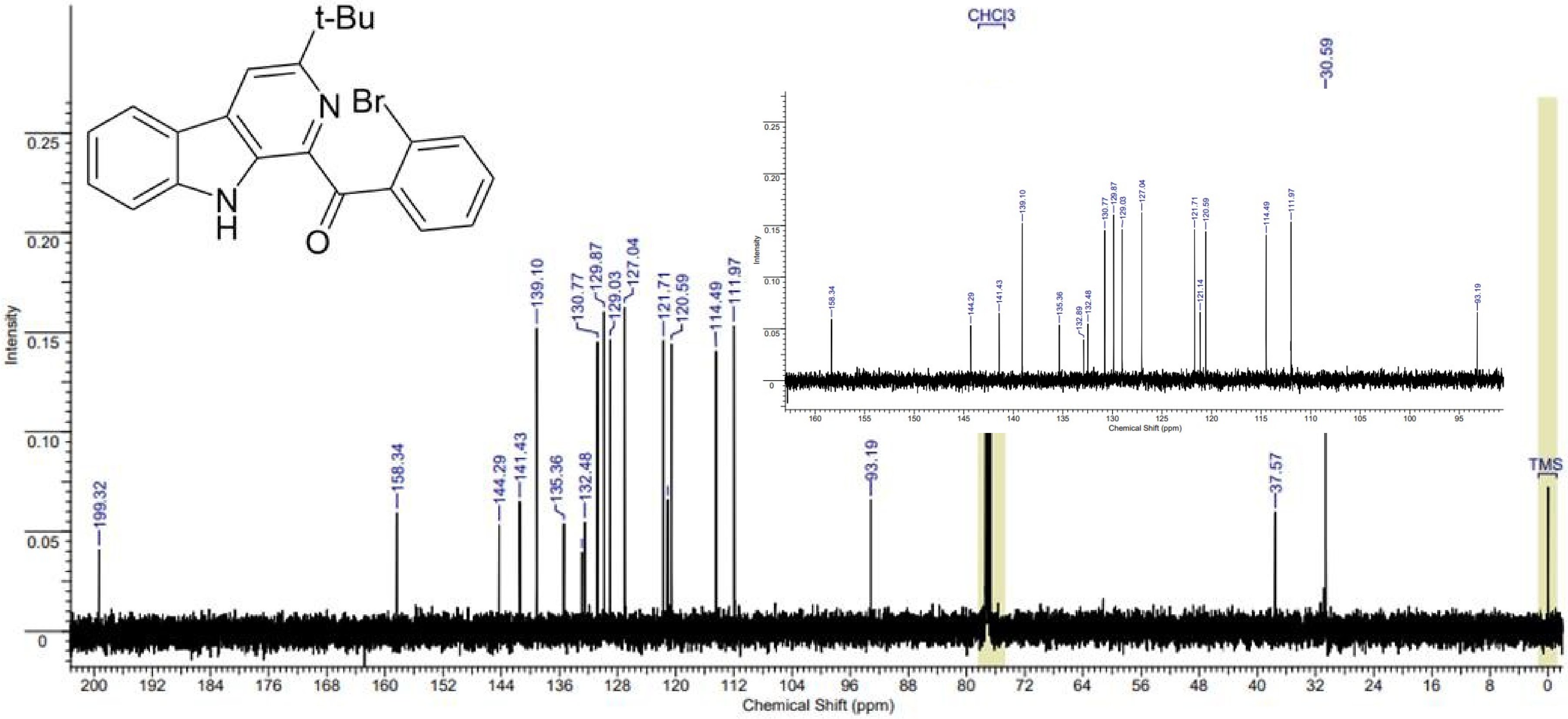

^{1}H NMR spectra of 1-(2′-iodobenzoyl)-3-*tert*-butyl-β-carboline

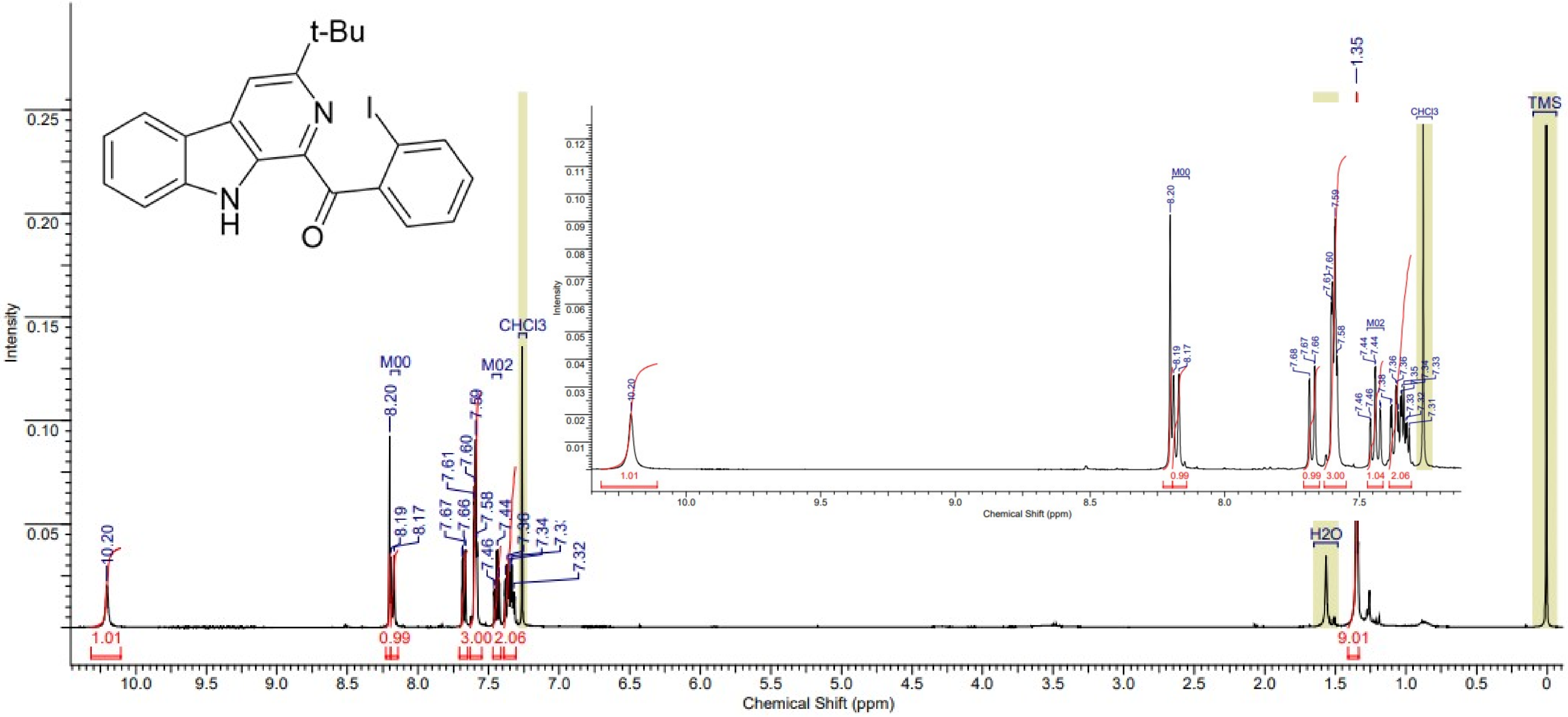

^{13}C NMR spectra of 1-(2′-iodobenzoyl)-3-*tert*-butyl-β-carboline

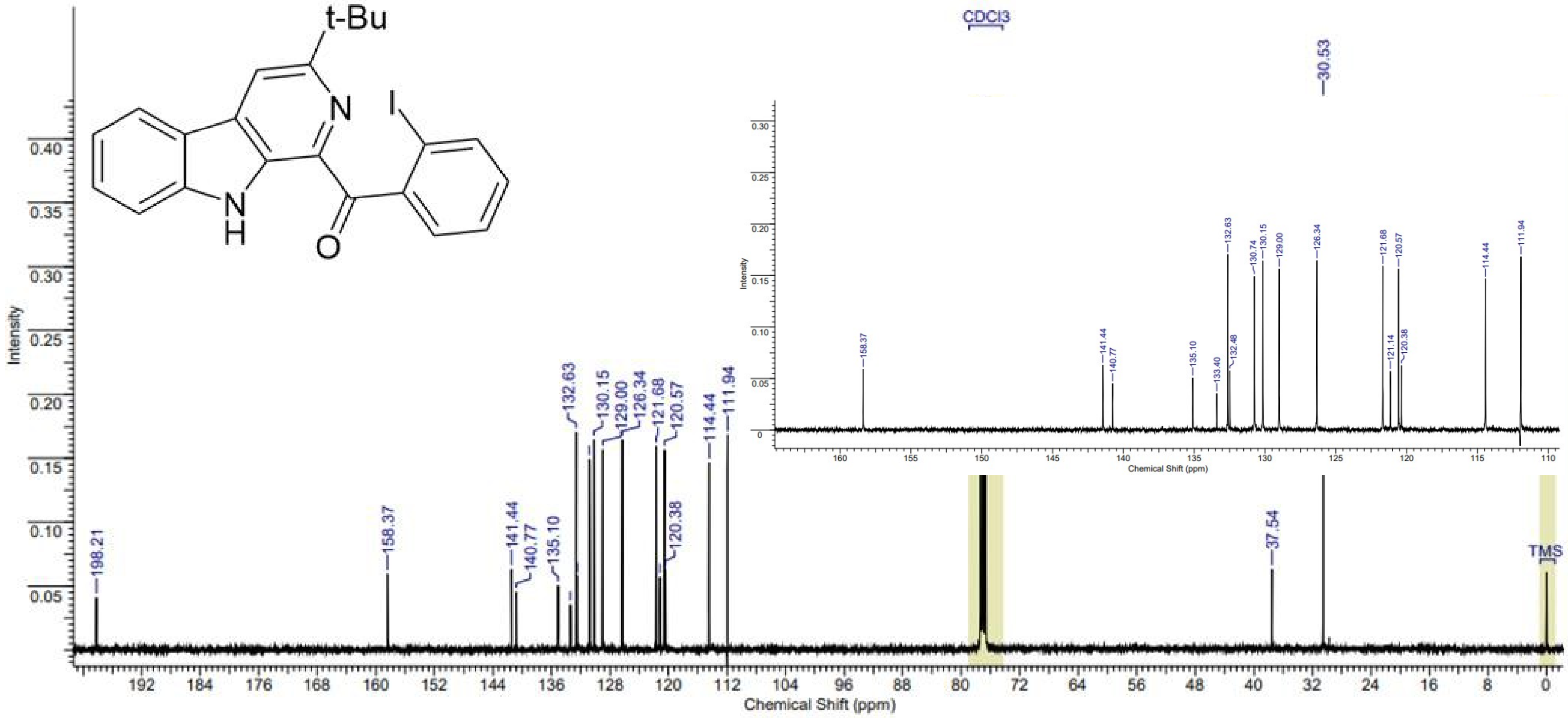

^1H NMR spectra of 12,13-dihydro-9-benzyloxy-13-oxopyrido[1,2-a:3,4-b′]diindol-5-ium

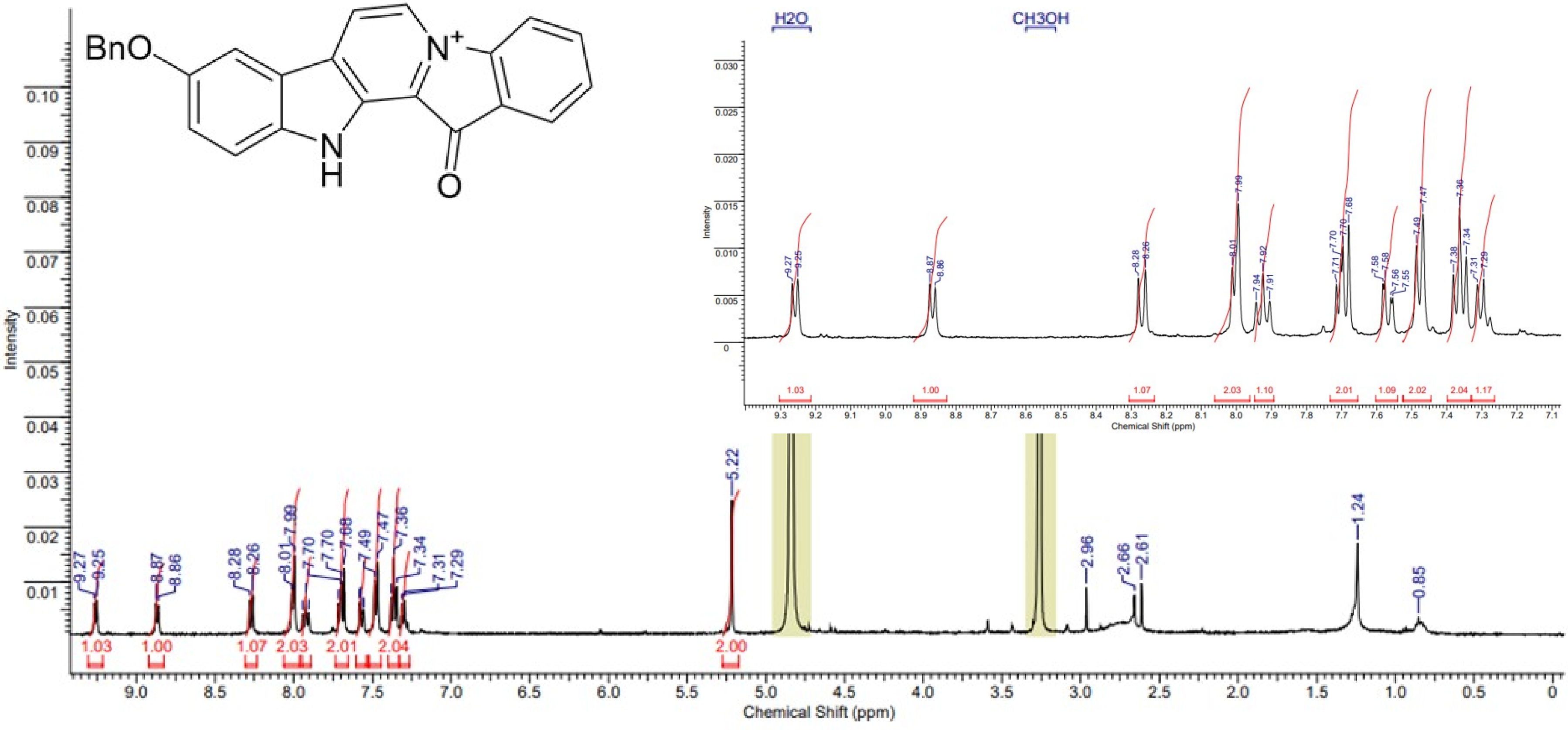

^{13}C NMR spectra of 12,13-dihydro-9-benzyloxy-13-oxopyrido[1,2-a:3,4-b′]diindol-5-ium

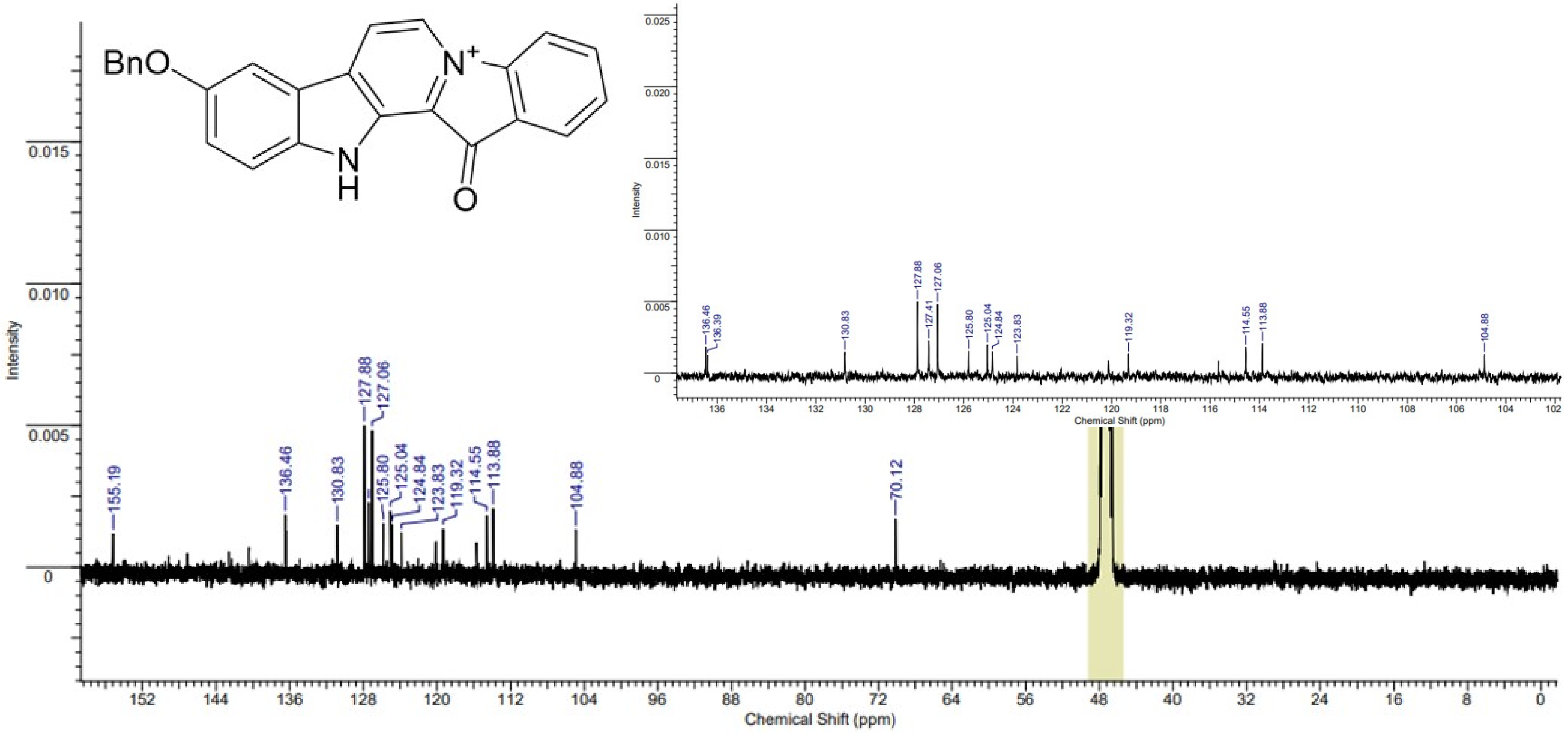

^{1}H NMR spectra of 12,13-dihydro-6-*tert*-butyl-13-oxopyrido[1,2-a:3,4-b′]diindol-5-ium

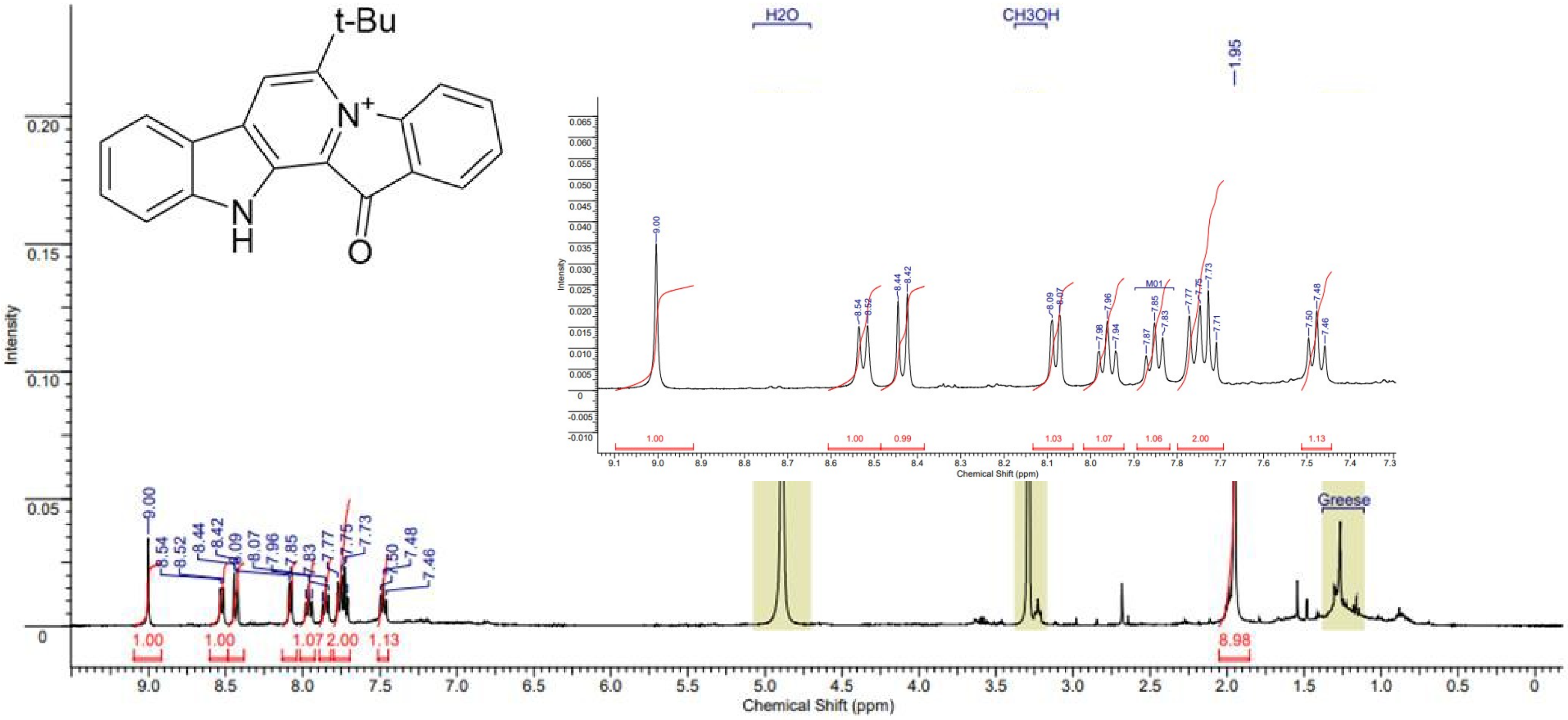

^{13}C NMR spectra of 12,13-dihydro-6-*tert*-butyl-13-oxopyrido[1,2-a:3,4-b′]diindol-5-ium

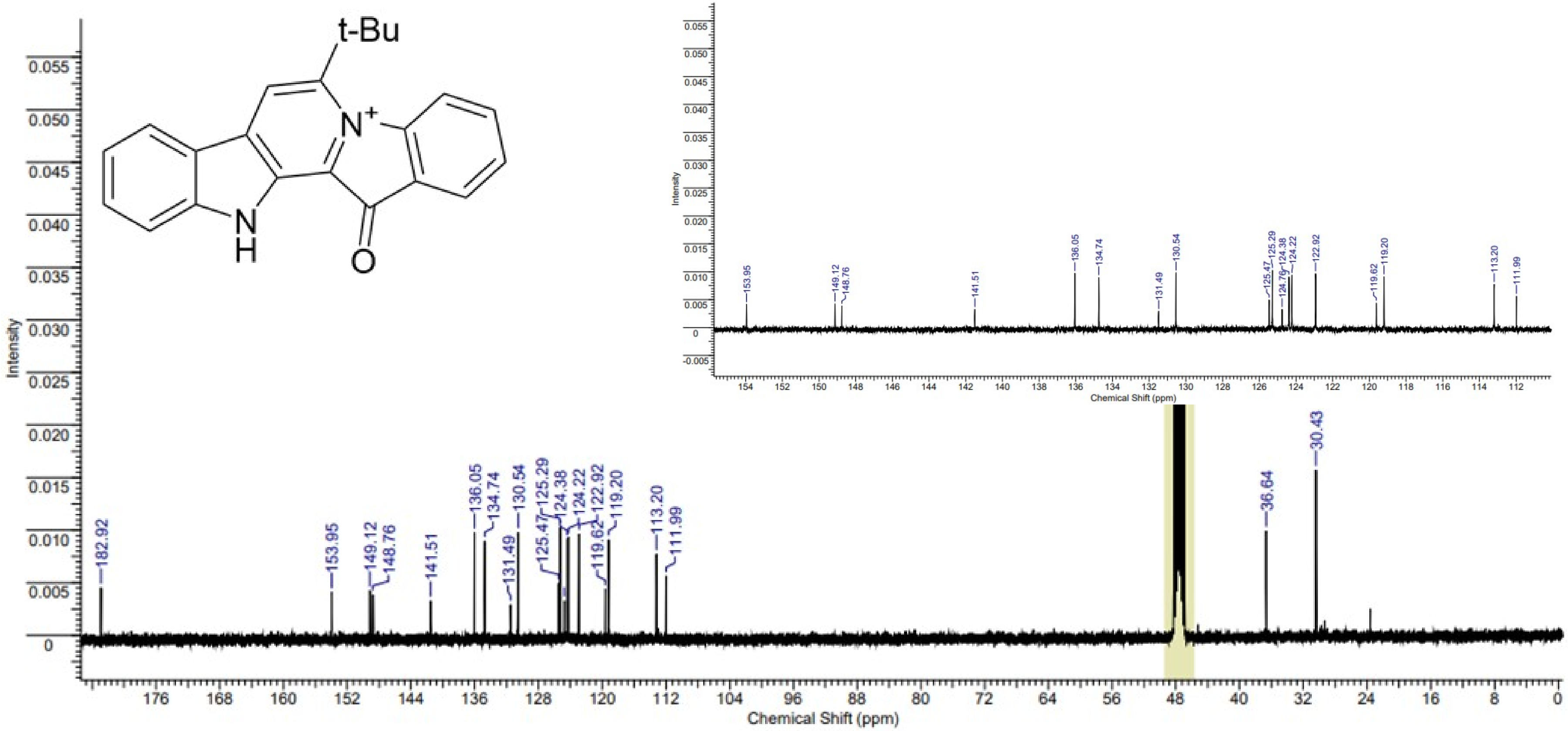

Supplement: Supplementary file 1 [file marinedrugs-21-00424-s001.zip › marinedrugs-2460097-supplementary.pdf]
